# Supplementary material for: Modeling tumor dynamics and predicting response to chemo-, targeted-, and immune-therapies in a murine model of pancreatic cancer
Source: bioRxiv. 2025 Jan 3:2025.01.03.631015. Preprint. [Version 1] doi: 10.1101/2025.01.03.631015 (PMC11722293; doi:10.1101/2025.01.03.631015)
Supplement: Supplement 1 [file NIHPP2025.01.03.631015v1-supplement-1.pdf]

bioRxiv preprint doi: https://doi.org/10.1101/2025.01.03.631015; this version posted January 3, 2025. The copyright holder for this preprint (which was not certified by peer review) is the author/funder, who has granted bioRxiv a license to display the preprint in perpetuity. It is made available under aCC-BY 4.0 International license.

Supplementary Material

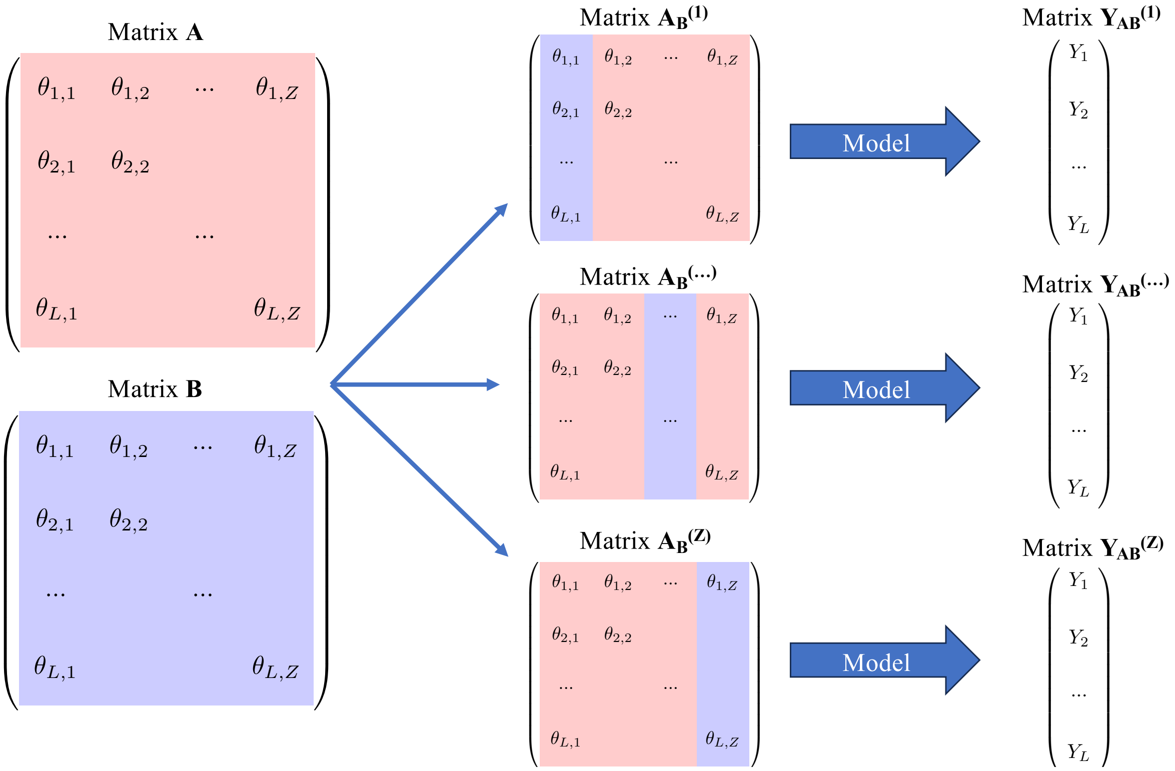

Supplemental Figure 1. Visual representation of total sensitivity index estimation using matrix notion. Theta, $\boldsymbol{\theta}$, is defined as a vector of parameters for a particular model. We began by developing matrices $\boldsymbol{A}$ and $\boldsymbol{B}$, where arbitrary column z is a vector of L values randomly generated from the sample space of the zth parameter. $\boldsymbol{A}$ and $\boldsymbol{B}$ are then used as components of a new hybrid matrix, with one column from $\boldsymbol{B}$ and the rest from $\boldsymbol{A}$. Then, the model is propagated for each hybrid matrix and for matrix $\boldsymbol{A}$. Variations between the output of matrix $\boldsymbol{A}$ compared to each hybrid matrix allow insight of parameter influence on model output, within the sampling space.
